# A predictive model of perceived stress during the first wave of the COVID-19 pandemic in university students Ecuadorians

**DOI:** 10.3389/fpsyt.2023.1202625

**Published:** 2023-07-13

**Authors:** Geovanny Genaro Reivan Ortiz, Rafael Yanza, Ximena Campoverde, Johanna Paulina Estrada Cherres, Lourdes Priscila Reinoso García, José Diaz, Roser Granero Pérez

**Affiliations:** ^1^Laboratory of Basic Psychology, Behavioral Analysis and Programmatic Development (PAD-LAB), Catholic University of Cuenca, Cuenca, Ecuador; ^2^Catholic University of Cuenca, Cuenca, Azuay, Ecuador; ^3^Autonomous University of Barcelona, Barcelona, Catalonia, Spain

**Keywords:** perceived stress, COVID-19, procrastination, emotional regulation, Ecuadorians

## Abstract

**Background and objectives:**

The situation caused by the confinement due to the COVID-19 pandemic and the mobility restriction implemented by governments worldwide had a significant impact on people’s routines. Stressors are known to increase emotional imbalance, uncertainty, and frustration in the general population. This study explores the factors that predispose to the risk of perceived stress from COVID-19 and determines the underlying mediating mechanisms in the Ecuadorian population.

**Method:**

The cross-sectional study an incidental non-probabilistic sample of *n* = 977 participating student volunteers from the four regions of the Republic of Ecuador (68.6% women and 31.4% men). Data on emotional regulation (ERQ), perceived stress (PSS), active procrastination (APS), diagnosis and symptoms related to COVID-19, social isolation, coexistence, and a sociodemographic questionnaire (biological sex, marital status, and age) were recruited. Statistical analysis was based on a structural equation model.

**Results:**

The risk of suffering perceived stress in the COVID-19 pandemic was higher for single women who have lived longer in social isolation, have lived with more people, have poor emotional regulation and high rates of procrastination. This structural model is similar in all Ecuadorian regions *χ*^2^ = 21.54 (*p* = 0.760), RMSEA = 0.001 (95%CI, 0.00–0.02), CFI = 0.998; TLI = 0.999; SRMR = 0.020.

**Discussion:**

Although our findings are consistent and revealing for the scientific community, the lack of discrimination of the data due to strict isolation measures, taken at different periods by the Ecuadorian government against positive cases of COVID-19, is discussed. The research was applied to the university population, it would be representative to extend the study to schools and colleges.

**Conclusion:**

We consider this work as a starting point for the creation of preventive models against perceived stress in the university environment in the event of health emergencies.

## 1. Introduction

In December 2019, health authorities alerted a new variant of the coronavirus family, SARS-CoV-2, in Hubei Province, China (1, 2). However, most cases affected by this virus present mild symptoms similar to those of a common cold. Therefore, a fraction of those infected present more severe symptoms that can lead to a severe acute respiratory syndrome, pneumonia, renal failure, and even death (3). This new variant of the virus is transmitted from person to person by air through secretions or respiratory droplets expelled as aerosols (contact with fomites), which facilitates its rapid spread (4). On February 11, 2020, the World Health Organization (WHO) named this pathogenic virus COVID-19 and promoted several containment measures focused on personal hygiene habits, ventilation of private and public areas, and social distancing (5).

Since its inception, COVID-19 has had a significant impact on society, with substantial adverse effects on physical and mental health (both of those directly infected and the rest of the general population) (6–8). Government authorities worldwide have issued various measures to encourage people to leave their homes as little as possible and encourage teleworking (9). Consequently, the population has been driven to adopt new lifestyles, which in some cases has involved the use of negative coping strategies to mitigate the perceived stressful situation, resulting in the emergence and aggravation of psychological problems (10–12).

Currently, worldwide so far, in 2022, there are approximately 442 million confirmed cases, 5 million deaths, and a total of 10 billion doses of vaccines administered (13). In Ecuador, the approximate calculation of these positive cases is 800,000, with 35,000 deaths and 13 million doses of vaccines administered (78.08% of the population fully vaccinated) (14). Emergency preparedness and response at the onset of the COVID-19 pandemic have been insufficient. Neither Ecuador nor any other country in the world was prepared for a pandemic of this type. The health crisis and the impacts on the Ecuadorian population during 2020 and 2021 have severely affected the national public health system and emergency response; still, they have also had socioeconomic, equity, and ethical dimensions in the country (9). Ecuador’s National Health System has been severely overwhelmed by the COVID-19 pandemic despite the efforts of the National Ministry of Public Health; this was primarily due to inadequate emergency health planning responses. The provinces of Pichincha, Guayas, Manabí, Azuay, El Oro, and Tungurahua were the most affected by the COVID-19 pandemic, with thousands of positive cases (15).

One of the effects of COVID-19 has been to increase the perceived stress levels of the individuals, leading to a worsening of anxiety and depressive symptoms (16, 17). Perceived stress is defined as a process of defensive response to a stimulus or pressure (which can be either positive or negative), and states of physical and emotional tension characterize it. It involves activation of the hypothalamic–pituitary–adrenal axis with activation of corticosteroids and the autonomic nervous system (18). Low levels of perceived stress are not harmful since they are a consequence of the need for readjustment of the organism. Pathological stress produces an imbalance between the contextual demands and the response capacity of the subjects to face them. The emitted response results from overexertion that leads to situations of exhaustion, fatigue, psychosomatic states, and other psychopathological alterations (19). The dominant explanatory theory of stress is based on a bio-psycho-social model. A person perceives a situation as threatening and emits a global response that is excessive and exceeds their capacity for resistance (20). In these situations, some subjects may use maladaptive coping strategies that lead to psychological distress (12, 21–26).

Several studies relate increased perceived stress levels to emotional dysregulation (27), understood as the lack of control over behavior, personal motivations, affect, and difficulty returning to a calm state. One of the dimensions most often related to stress states is expressive suppression because it relates to emotional exhaustion (28).

Another stress-related maladaptive behavior observed is procrastination (29–33). As is known, stress-induced discomfort situations cause activities to be postponed or not completed. Precisely on this point, the confinement by COVID-19 has led the individuals to postpone activities and avoid situations that were part of their daily life, such as work, social activities, and studies (34). As a result, many students and workers have been forced to be absent from school and work (35, 36).

Sociodemographic studies recorded during the confinement phase due to COVID-19 have identified variables with predictive capacity for perceived stress, such as female sex (39.40), single marital status (37), isolation time (38), diagnosis of COVID-19, symptoms associated with COVID-19 (39) and population regions (15).

Studies in Latin America (37–39) have reported high-stress cases due to the COVID-19 pandemic. Precise Ecuador was one of the countries most affected by the pandemic (report of more than 65,000 cases of people infected by COVID-19) (40) with deplorable financial resources and incomplete health facilities. Ecuador has had severe difficulties determining possible cases of infection, containing its spread, and treating patients (41, 42). This critical situation, wrapped in a context of significant vulnerability, has created a feeling of extreme helplessness among the population, affecting mental health (43), especially generating perceived stress.

In summary, the control measures promoted worldwide to control the spread of the COVID-19 pandemic (characterized by the accentuation of social distance and home isolation) have substantially impacted people’s lifestyles, increasing the risk of unhealthy behavioral patterns (44). For example, disruption of the daily routine of work and studies, or being continuously exposed to news about the infection provided by the media, could have increased levels of uncertainty and frustration in the individuals, leading to increases in perceived stress levels (7, 10, 37, 38). Therefore, identifying the underlying mechanisms that mediate the level of perceived stress and knowing if these mechanisms are different in the regions evaluated is necessary to develop future actions in the face of new waves of the COVID-19 pandemic or other situations potentially generating high pressure on individuals. The objective of this study is to obtain mediation models that evaluate the direct and indirect effects (mediation relationships) between the following variables: socio-demographic (ecuadorian region, marital status: single), contextual to COVID-19 confinement, procrastination, and emotional regulation on the levels of stress perceived by university students in Ecuador, during the time of mandatory isolation (period March–September 2020), where there was no presence of vaccines against the virus.

According to the available scientific evidence, a model has been proposed, whose hypotheses represented in the direct associations are: The female sex acts as a direct predictor of symptoms of COVID-19 (34). Greater symptoms related to COVID-19 increase procrastination (35). Single marital status predicts: COVID-19 disease (34), procrastination (45), and emotional regulation dimensions (cognitive reappraisal and expressive suppression) (46). Older years of age increase cognitive reappraisal (47). Higher levels of procrastination (29–31, 33) and higher levels of the dimensions of emotional regulation (cognitive reappraisal and expressive suppression) (27, 28) increase perceived stress. Finally, the composition of these relationships is different in the Ecuadorian regions evaluated (15).

## 2. Method

### 2.1. Participants

The non-probabilistic sample consisted of *n* = 977 volunteer university students from the four regions that are part of the Republic of Ecuador (68.6% women and 31.4% men): Insular *n* = 10 (1.01%), Coast *n* = 291 (29.79%), Highlands *n* = 597 (61.11%) and West *n* = 79 (8.09%). The students came from five representative universities in the country, relevant to the north (Central de Quito and Técnica de Ibarra) and the south (Técnica de Machala, Santiago de Guayaquil, and Católica de Cuenca), in areas related to health (medicine, dentistry, psychology, and nursing) and intermediate level of study (from fifth and sixth academic semester). Considering that many of the students received their classes at their homes telematically, the territorial location where the data were collected depends on the geographic region where they would receive their computer classes remotely. Therefore, making up the urban area where the universities are located and the rural area of their homes. Sampling was incidental due to accessibility. Inclusion criteria considered university students in the Ecuadorian regions who had signed the informed consent form were between 18 and 25 years of age, were in home isolation, and had access to a computer or specialized service to answer the survey. None of the participants reported having any medical or organic condition that prevented them from answering the tests, as well as having any anxiety, depression or psychiatric comorbidity 6 months before the evaluation. The distribution of participants according to their sociodemographic, clinical, and pandemic variants for each region is reported in Table 1.

**Table 1:** Description of the sample.

		Range (Min. – Max.)	Total		Insular		Coast		Mountain		West				
			*n* = 977		*n* = 10		*n* = 291		*n* = 597		*n* = 79				
*Sociodemographics*			*n*	%	*n*	%	*n*	%	*n*	%	*n*	%	*χ* ^2^	*df*	*p*
Sex															
Women			670	68.6%	7	70.0%	220	75.6%	388	65.0%	55	69.6%	10.27	3	0.016*
Men			307	31.4%	3	30.0%	71	24.4%	209	35.0%	24	30.4%			
Marital															
Single			869	88.9%	7	70.0%	266	91.4%	524	87.8%	72	91.1%	11.23	6	0.082
Married			88	9.0%	3	30.0%	23	7.9%	56	9.4%	6	7.6%			
Divorced			20	2.0%	0	0.0%	2	0.7%	17	2.8%	1	1.3%			
			Mean	SD	Mean	SD	Mean	SD	Mean	SD	Mean	SD	*F-stat*	*df*	*p*
Age (yrs-old)		12 (17–29)	20.13	2.62	20.40	4.58	20.09	2.58	20.14	2.60	20.20	2.69	0.08	3; 973	0.971
*Context during COVID-19*			*n*	%	*n*	%	*n*	%	*n*	%	*n*	%	*χ* ^2^	*df*	*p*
Symptoms															
No			479	49.0%	10	100.0%	103	35.4%	325	54.4%	41	51.9%	39.29	3	<0.001*
Yes			498	51.0%	0	0.0%	188	64.6%	272	45.6%	38	48.1%			
Illness															
No			937	95.9%	10	100.0%	273	93.8%	577	96.6%	77	97.5%	5.00	3	0.164
Yes			40	4.1%	0	0.0%	18	6.2%	20	3.4%	2	2.5%			
Social isolation															
No			586	60.0%	5	50.0%	166	57.0%	363	60.8%	52	65.8%	2.75	3	0.431
Yes			391	40.0%	5	50.0%	125	43.0%	234	39.2%	27	34.2%			
Living with others															
No			26	2.7%	0	0.0%	6	2.1%	19	3.2%	1	1.3%	1.90	3	0.594
Yes			951	97.3%	10	100.0%	285	97.9%	578	96.8%	78	98.7%			
*Questionnaires*	α		Mean	SD	Mean	SD	Mean	SD	Mean	SD	Mean	SD	*F-stat*	*df*	*p*
ERQ-cognitive reappraisal	0.81	36 (6–42)	25.05	8.23	23.20	10.26	26.25	7.92	24.73	8.28	23.29	8.27	3.79	3; 973	0.010*
ERQ-emotion suppression	0.77	24 (4–28)	17.67	6.37	17.10	7.36	18.36	6.06	17.46	6.44	16.77	6.75	1.91	3; 973	0.126
Procrastination	0.88	105 (0–105)	52.35	18.67	51.20	25.51	53.79	18.25	51.64	18.64	52.56	19.58	0.88	3; 973	0.449
Perceived stress	0.83	16 (4–20)	13.01	2.43	12.90	3.81	13.21	2.20	12.92	2.44	12.91	2.87	1.01	3; 973	0.386

df, degrees of freedom; SD, standard deviation; EQR, Emotional Regulation Questionnaire; α, Cronbach’s alpha. *Bold: significant comparisons.

### 2.2. Instruments

*Emotional Regulation Questionnaire (ERQ)* (48). Individual differences in emotional regulation processes: implications for affection, relationships, and well-being. The questionnaire is composed of 10 items in which the individual must express their degree of agreement about how they habitually regulate their emotions. The participant responds according to a seven-point Likert-type scale, ranging from 1 (strongly disagree) to 7 (strongly agree). The first factor collects the scores that assess cognitive reappraisal. The second factor refers to emotional suppression. A higher score indicates a higher level of cognitive reappraisal and/or emotional suppression. The Spanish version of the instrument was used in the present study (49). Cronbach’s alpha indices for our study sample are reported in the results (Table 1).

*Perceived Stress Scale (PSS)* (50). A self-report instrument that assesses the level of stress perceived during the last month. It contains 14 items with a 5-point Likert-type response scale (0 = never, 1 = almost never, 2 = occasionally, 3 = often, 4 = very often). A higher score indicates a higher level of perceived stress. The Spanish version of the instrument was used in the present study (49). Cronbach’s alpha indices for our study sample are reported in the results (Table 1).

*Active Procrastination Scale (APS)* (51) is a 16-item scale based on the underlying components of active procrastination. It is a 7-point Likert-type scale with a response format ranging from 1 (not true at all) to 7 (absolutely true). A higher score indicates a higher level of procrastination. The Spanish version of the instrument was used in the present study (49). Cronbach’s alpha indices for our study sample are reported in the results (Table 1).

*Sociodemographic questionnaire*. It consists of a short, structured, closed-response survey containing sociodemographic questions (biological gender, marital status, and age measured in years).

*Pandemic questionnaire*. Four exploration questions with two answer options (Yes/No) developed by principal investigators based on previous study (52). It consists of questions about the pandemic context: symptoms related to COVID-19, diagnosis of COVID-19, social isolation and coexistence (the questions were interpreted dichotomously absence = 0 and presence = 1).

### 2.3. Process

Data were collected from March to September 2020 in the Republic of Ecuador. The participants were amid social isolation due to COVID-19 and conducted their classes telematically by videoconference.

The study was conducted under the guidelines of the latest Declaration of Helsinki for research on human subjects (53) and was approved by the Ethics Committee for Research on Human Subjects of the Universidad UTE (IRB-UTE /CEISH UTE) approval code: IMP-*SIC*-LLA CUIO 1408 20, and had the bioethics authorization of the university academic council: Universidad Central de Quito, Universidad Santiago de Guayaquil, Universidad Técnica de Ibarra, Universidad Técnica de Machal, a and Universidad Católica de Cuenca.

Approximately 25,000 students from the five universities received an email invitation to participate in the study voluntarily and free of charge, of which 977 students who agreed to participate gave informed consent and were sent an online survey conducted through the Google Forms^™^ program.

## 3. Data analysis

Statistical analysis was performed with Stata17 for Windows (54). Comparisons between groups were based on the chi-square test (χ^2^) for categorical variables and analysis of variance (ANOVA) for quantitative measures. Path analyses implemented through structural equation modeling (SEM) assessed direct and indirect effects. That included mediational links between sociodemographic variables (gender, marital status, and age), contextual and individual variables during COVID-19 confinement (social isolation, presence of psychological and/or physical symptoms, and the use of treatment for mental or physical illness). It also included measures of emotional regulation (ERQ scores), procrastination, and perceived stress. Path analysis procedures are a direct extension of multiple regression models (55) and can be used for both exploratory and confirmatory modeling (which allows testing and development of theories) (56). This work used the maximum likelihood estimation (MLE) parameter estimation method, and goodness-of-fit was evaluated. That is, using standard statistical measures: χ^2^ test, root mean square error of approximation (RMSEA), Bentler comparative fit index (CFI), Tucker-Lewis index (TLI), and standardized root mean square residual (SRMR). The adequate model fit was considered non-significant in 2 tests and if the following criteria were met (57): RMSEA<0.08, TLI > 0.9, CFI > 0.9 and SRMR<0.1.

In this study, we have used the Finner’s-method to control Type-I error due to multiple statistical analyses. This is a familywise error rate stepwise procedure, which offers a more powerful capacity than the classical Benforroni’s correction (58).

## 4. Results

### 4.1. Sample characteristics

Table 1 shows the description of the participants in the study, and the psychological scales have good psychometric properties. The majority of participants were female (*n* = 670, 68.6%) and single (*n* = 869, 88.9%). The mean age was 20.13 (SD = 2.62). Regarding contextual variables during COVID-19 confinement, more participants reported the presence of physical or psychological symptoms (*n* = 498, 51.0%), lack of treatment for physical or mental illness (*n* = 937, 95.9%), lack of social isolation (*n* = 586, 60.0%), and cohabitation (*n* = 951, 97.3%). The comparison between geographic areas achieved statistical differences for the sex of the participants (higher proportion of women among the Coast group), the presence of symptoms during the confinement (higher proportion among the Island group), and the ERQ cognitive reappraisal scale (higher mean among the participants among the Coast group).

### 4.2. Mechanisms that explain the level of perceived stress during confinement: trajectory analysis

Table 2 shows the correlation matrix for the variables considered in the study. As a result of the strong association between sample size and the results of the null hypothesis tests for the correlation model, only coefficients within the ranges mild–moderate (|R|> > 0.24) to large-high (|R|> > 0.37) were considered as relevant. Perceived stress levels positively correlated with higher scores on the ERQ (emotional regulation questionnaire) and procrastination scales. The ERQ scales also correlated very positively.

**Table 2:** Correlation matrix.

		2	3	4	5	6	7
1	Age (yrs-old)	−0.031	0.006	0.070	0.037	0.027	0.010
2	COVID-isolation	–	0.031	0.015	0.015	0.026	0.003
3	COVID-living with others		–	−0.045	−0.017	−0.011	−0.049
4	ERQ-cognitive reappraisal			–	**0.772**	0.140	**0.300**
5	ERQ-emotion suppression				–	0.126	**0.311**
6	Procrastination					–	**0.310**
7	Stress						–

Bold: statistically significant correlation.

**Figure 1:**
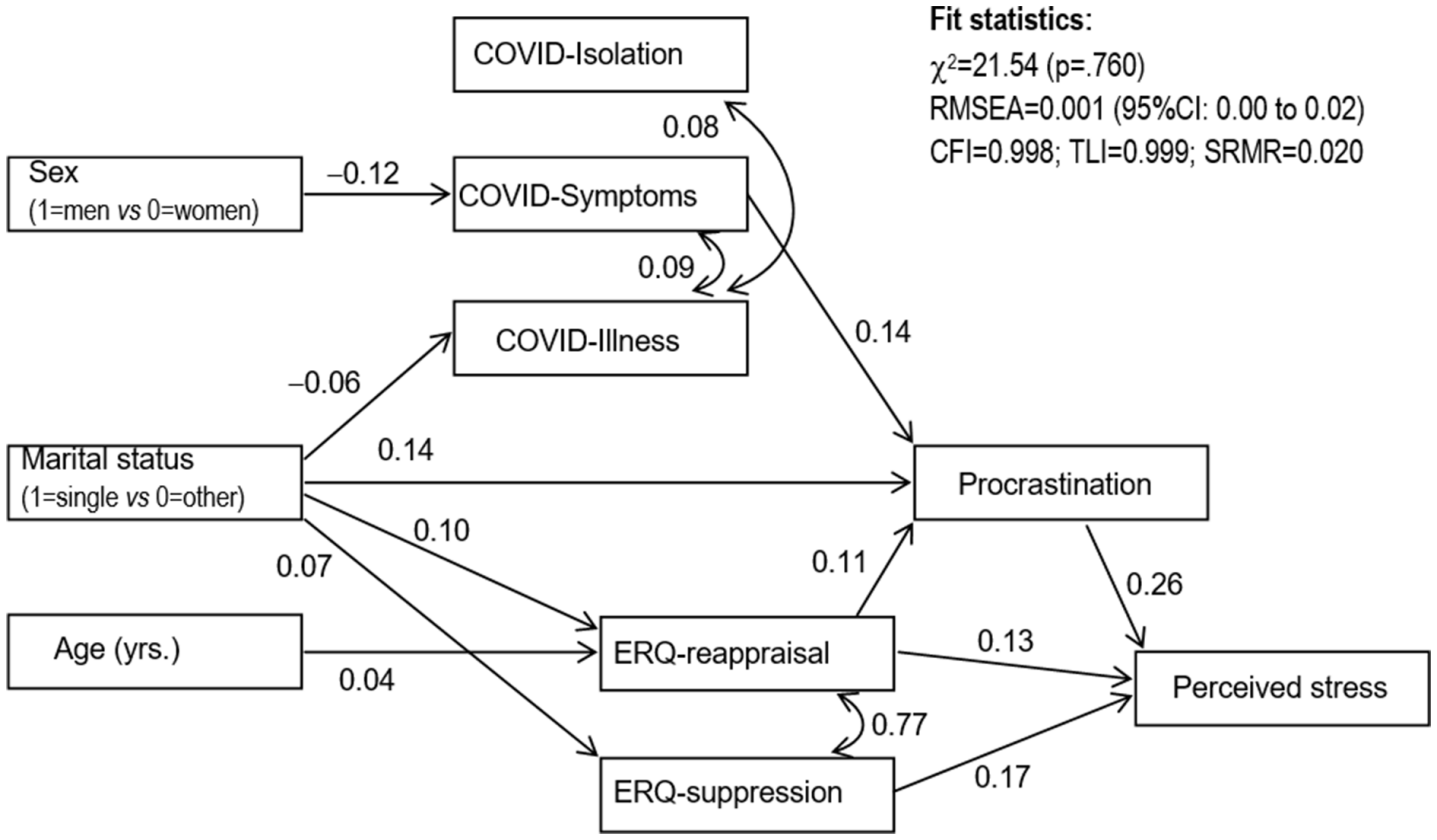
Diagram of the trajectory plot obtained in the SEM for stress: standardized coefficients. Only the significant coefficients remained in the final model. Sample size: *n* = 977.

Figure 1 shows the path diagram with the standardized coefficients (Table 3 contains the complete results of the model: direct, indirect, and total effects). For the categorical variables, we have included into brackets the code used with the aim to allow the interpretation of the coefficients. This SEM selected in the study as the optimal model for the data set retained only significant associations. An adequate fit was achieved: *χ*^2^ = 21.54 (*p* = 0.760), RMSEA = 0.001 (95% confidence interval: 0.00–0.02), CFI = 0.998, TLI = 0.999 and SRMR = 0.020.

**Table 3:** Results obtained in the path analysis.

		Direct effects						Indirect effects						Total effects					
		Coeff.	SE	z-stat	*p*-value	95%CI (coeff)		Coeff.	SE	z-stat	*p*-value	95%CI (coeff)		Coeff.	SE	z-stat	*p*-value	95%CI (coeff)	
COVID-isolation	Sex (1 = male vs. 0 = female)	−8.636	5.587	−1.55	0.122	−19.587	2.315	0.000	*No.path*					−8.636	5.587	−1.55	0.122	−19.587	2.315
COVID-symptoms	Sex (1 = male vs. 0 = female)	−0.133	0.034	−3.89	0.000	−0.199	−0.066	0.000	*No.path*					−0.133	0.034	−3.89	0.000	−0.199	−0.066
ERQ-reappraiss	Age (yrs-old)	0.132	0.063	2.08	0.038	0.008	0.256	0.000	*No.path*					0.132	0.063	2.08	0.038	0.008	0.256
	Single	2.943	0.832	3.54	0.000	1.312	4.574	0.000	*No.path*					2.943	0.832	3.54	0.000	1.312	4.574
Procrastination	COVID-symptoms	4.950	1.161	4.26	0.000	2.675	7.225	0.000	*No.path*					4.950	1.161	4.26	0.000	2.675	7.225
	ERQ-reappraiss	0.276	0.071	3.89	0.000	0.137	0.415	0.000	*No.path*					0.276	0.071	3.89	0.000	0.137	0.415
	Sex (1 = male vs. 0 = female)	0.000	*No.path*					−0.656	0.228	−2.88	0.004	−1.104	−0.209	−0.656	0.228	−2.88	0.004	−1.104	−0.209
	Age (yrs-old)	0.000	*No.path*					0.036	0.020	1.83	0.067	−0.003	0.075	0.036	0.020	1.83	0.067	−0.003	0.075
	Marital (1 = single vs. 0 = other)	8.481	1.862	4.55	0.000	4.831	12.130	0.812	0.310	2.62	0.009	0.203	1.420	9.293	1.865	4.98	0.000	5.638	12.947
Stress	COVID-symptoms	0.000	*No.path*					0.174	0.045	3.87	0.000	0.086	0.262	0.174	0.045	3.87	0.000	0.086	0.262
	ERQ-reappraiss	0.036	0.014	2.63	0.009	0.009	0.062	0.010	0.003	3.58	0.000	0.004	0.015	0.045	0.014	3.30	0.001	0.018	0.072
	Procrastination	0.035	0.004	9.20	0.000	0.028	0.043	0.000	*No.path*					0.035	0.004	9.20	0.000	0.028	0.043
	ERQ-suppression	0.070	0.017	4.03	0.000	0.036	0.104	0.000	*No.path*					0.070	0.017	4.03	0.000	0.036	0.104
	Sex (1 = male vs. 0 = female)	0.000	*No.path*					−0.023	0.008	−2.74	0.006	−0.039	−0.007	−0.023	0.008	−2.74	0.006	−0.039	−0.007
	Age (yrs-old)	0.000	*No.path*					0.006	0.003	1.76	0.079	−0.001	0.013	0.006	0.003	1.76	0.079	−0.001	0.013
	Marital (1 = single vs. 0 = other)	0.000	*No.path*					0.538	0.110	4.91	0.000	0.323	0.753	0.538	0.110	4.91	0.000	0.323	0.753
ERQ-suppression	Marital (1 = single vs. 0 = other)	1.529	0.648	2.36	0.018	0.259	2.799	0.000	*No.path*					1.529	0.648	2.36	0.018	0.259	2.799
COVID-illness	Marital (1 = single vs. 0 = other)	−0.038	0.020	−1.87	0.061	−0.077	0.002	0.000	*No.path*					−0.038	0.020	−1.87	0.061	−0.077	0.002

Coeff., non-standardized coefficient; SE, standard error; 95%CI, 95% confidence interval.

According to the hypotheses raised, it is confirmed by a causal model that: the female gender acts as a direct predictor of symptoms of COVID-19; Similarly, single marital status influences the increase in symptoms associated with COVID-19. Years of age directly influence cognitive reappraisal; procrastination and emotional regulation dimensions act as direct predictors of perceived stress. In other words, higher scores on the procrastination dimension directly predicted higher stress levels, and more difficulties were found in emotional regulation strategies (higher scores on the ERQ cognitive reappraisal and expressive suppression). It also observed different indirect (mediational) links explaining the measure of perceived stress. First, procrastination was a mediating variable in different ways: (a) Being female increased the likelihood of symptoms during confinement, which increased procrastination and thus perceived stress levels. (b) Being single also directly increased procrastination and, therefore, stress score. (c) Singleness and older age were predictors of higher scores on the cognitive reappraisal dimension of the ERQ, which increased procrastination and thus the likelihood of increased stress.

## 5. Discussion

The objective of this study was to evaluate the relevance of a predictive model of perceived stress and to know its differences between the regions of the Republic of Ecuador in the period from March to September 2020 in university students. The results suggest that time spent in confinement, social isolation, living with more people in one environment, non-assertively regulated emotions (expressive suppression), procrastination, and being a woman increase the severity of perceived stress. The structure of these relationships is similar in all Ecuadorian regions (Insular, Coast, Mountain and West).

Our results confirm the hypotheses studied in an integrated causal model. The data are consistent with reported studies on the influence of confinement time on perceived stress (27, 47). Similarly, social isolation is an aggravating factor in the course of stress in times of confinement by COVID-19 (27); with this, one can interpret that withdrawing from the social context increases a set of long-term psychopathological states (59), including stress (16–18, 60).

Although the results are consistent with our hypotheses, several studies mention that living with people in a family environment has many positive effects on mental health (61–63). This is possibly due to the particular characteristics of the pandemic in Ecuador, such as the strict social confinement in all regions of the country (64), presence of overcrowding in homes (65), effects of gender violence (sexism) (66), use of technologies for online learning (67), fear of getting infected (68), and economic problems derived from the health situation (69). Added to this, the characteristics of the social context in Ecuador are essential since most university students live with their families, and it is also common to live in extended families. This makes it possible that family problems have arisen during the pandemic and with the whole family staying at home.

On the other hand, our hypothesis that expressive suppression becomes the non-assertive emotional component that acts directly and mediates perceived stress is confirmed. This would indicate the clinical and psychopathological relevance of emotional regulation, as occurs in many psychological disorders (23, 24) such as depression (70, 71), bipolar disorder (72), generalized anxiety disorder (73, 74), social anxiety (75, 76), borderline personality disorder (77), eating disorders (78), and substance-related disorders (79). Therefore, the theoretical framework of transdiagnosis would explain the high rates of comorbidity that occur between different clinical disorders, emotional regulation being a key factor in their development and maintenance (80, 81).

These results indicate that emotion regulation has considerable overlap with perceived stress management. The studies strengthen our analysis of the role of managed emotional reactions in perceived stress that involve emotional regulation, such as expressive suppression and cognitive reappraisal (24, 82). This could be because when people face events, emotion regulation allows them to assess the emotional impact of the condition and helps determine what types of emotional reactions are appropriate, as well as when and how they express emotions, as might have occurred in the pandemic by COVID-19 (83).

Empirical evidence has shown that procrastination is one of the most frequent stressors presented by confinement, revealing that procrastination is associated with poorer psychological health (84–86). The results of the study corroborate our hypothesis, indicating that a greater tendency to procrastinate is related to the presence of greater stress. Despite the fact that studies have reported the association of anxiety and depression associated with procrastination (83, 87–89), in our study these variables were not considered because our model was to know the direct prediction of perceived stress, integrating emotional regulation, procrastination and contextual variables related to COVID-19.

A recent study revealed that female college students, compared to males, have higher levels of perceived stress due to the COVID-19 lockdown (71, 90–93). Our hypothesis corroborates these results. Possibly, these findings were due to the fact that the composition of the sample was made up of young university students. In the same way, we believe that these results may be influenced by a greater proportion of women in our study sample, statistically differentiated in each Ecuadorian region, so we believe that the results should be interpreted with caution.

Finally, despite the fact that our hypothesis was to find a different relationship model between regions (15), our results indicate the opposite. Possibly this is due to the fact that the composition of the sample is homogeneous among its characteristics (university profile).

Regarding the presence of symptoms related to COVID-19, the results indicated a statistically significant difference indicating high values in the Coast region. We believe that this result is due to the fact that most of the presence of deaths due to COVID-19 have been presented by the Coast region of Ecuador, as mentioned by several studies (9, 14, 15). Possibly this situation has influenced the presence of high values in the cognitive reappraisal of this region in a statistically significant way, as indicated by our results, which would have led its inhabitants to adopt an emotional regulation strategy that implies resignifying an event to change their perception. Emotional effect, this ability would have allowed them to reduce negative emotional experiences, denoting it as a protection factor against the health situation of COVID-19.

### 5.1. Strengths

The present study has three strengths: the study sample, the composition of the data collected in the natural course of the pandemic, and the statistical analysis. According to the first strength, the population was marked by the geographic regions of Ecuador, giving a sociocultural representation of the country made up of university students of middle cycles of academic careers related to the area of health who have access to the Internet through the modality online survey, allowing for specific external validity in the selected study population. The second strength included acquiring information on variables inherent to the measures established by the leaders of each country, representative of and in keeping with the natural context of health. Thirdly, path analysis involves the study of all the variables in the same model, allowing us to determine the direct and indirect effects of the study.

### 5.2. Limitations

Although our findings are consistent and revealing for the scientific community, however, there were limitations in conducting the study. One of them was the use of digital platforms to receive information, which probably hindered the natural response of the participant, as it could have been completed without supervision. One possibility to cover this bias was using self-applied paper instruments; however, this was not feasible given the current situation due to the pandemic. On the other hand, another fact that can be considered a limitation was the lack of discrimination of data taken in different periods since in Ecuador, there were times when there were high numbers of cases with a proven diagnosis, which forced the Emergency Operations Committee (COE by its Spanish acronym) of Ecuador to take strict isolation measures on several occasions. Finally, since this study is applied to the university population, schools and colleges would be left aside. Following studies along these lines may involve the different educational cycles to have a complete distribution of the results in the country. These deficiencies can be counterbalanced in future studies to provide novel reports to the scientific community. However, we consider this work a starting point for creating preventive models against perceived stress in the university setting in the face of health emergencies.

## 6. Conclusion

We concluded that being a woman, the time spent in confinement, social isolation, living with more people in one environment, not assertively regulating emotions (expressive repression), and procrastinating activities increased the risk of suffering perceived stress during the COVID-19 pandemic. The underlying model of relationships between the study variables to predict the severity of perceived stress is the same in the Ecuadorian regions evaluated.

## Data availability statement

The original contributions presented in the study are included in the article/Supplementary material, further inquiries can be directed to the corresponding author.

## Ethics statement

The studies involving human participants were reviewed and approved by CEISH-UCACUE is the Human Beings Research Ethics Committee Catholic University of Cuenca. The patients/participants provided their written informed consent to participate in this study.

## Author contributions

GR conducted the introduction and discussion of the study. RG developed the method and the results. RY, XC, JE, LR and JD recruited the data, reviewed and proofread the entire manuscript. All authors contributed to the article and approved the submitted version.

## Funding

RG was supported by the ICREA-Academy-2021 award.

## Conflict of interest

The authors declare that the research was conducted in the absence of any commercial or financial relationships that could be construed as a potential conflict of interest.

## Publisher’s note

All claims expressed in this article are solely those of the authors and do not necessarily represent those of their affiliated organizations, or those of the publisher, the editors and the reviewers. Any product that may be evaluated in this article, or claim that may be made by its manufacturer, is not guaranteed or endorsed by the publisher.
